# Application of Combined Superficial Femoral Artery Stenting and Deep Femoral Artery Profundoplasty in Chinese Patients With Lower Extremity Artery Disease

**DOI:** 10.3389/fsurg.2021.646978

**Published:** 2021-12-23

**Authors:** Guoqing Chi, Mingchao Ding

**Affiliations:** Department of Interventional Vascular Surgery, Aerospace Center Hospital, Beijing, China

**Keywords:** lower extremity artery disease, Rutherford grade, superficial femoral artery stenting, Chinese patients, deep femoral artery profundoplasty

## Abstract

**Objective:** Lower extremity artery disease (LEAD) increases sharply with age and results in severe burden in individuals and in society. This study aimed to compare the efficiency of simple superficial femoral artery stenting (SFAS) and the hybrid operation, such as combined SFAS and deep femoral artery profundoplasty (DFAP), in the treatment of Chinese patients with LEAD, classified as Rutherford grades 3–5.

**Methods:** There were 200 patients with LEAD classified as Rutherford grades 3–5 included in the simple SFAS group (*n* = 100) and the combined SFAS and DFAP group (*n* = 100).

**Results:** All the patients had median age of 71 years, and there were 143 males (71.5%). Not only the increase rate of ankle brachial index (ABI), but also reduction rate of Rutherford grade, were significantly higher after surgery in the combined SFAS and DFAP group than in the simple SFAS group (*P* < 0.05 for all). The patency rate of patients in the combined SFAS and DFAP group was significantly higher than that of patients in the simple SFAS group during the follow-up (*P* < 0.05). Proportion of amputation and claudication distance <200 m had no significant difference between the two groups during the 2-year follow-up (*P* > 0.05 for all).

**Conclusion:** This study demonstrated that, compared with SFAS, combined SFAS and DFAP improved not only the ABI and the Rutherford grade after surgery but also the patency rate during the follow-up in Chinese patients with LEAD. Hybrid operation has significant value in alleviating clinical symptoms after surgery, and, thereby, improving vascular prognosis in Chinese patients.

## Introduction

Arteriosclerosis accounts for 60–80% of lower extremity artery disease (LEAD), and results in severe burden in individuals and in society (1, 2). Balloon dilatation and stent placement of superficial femoral artery, namely, superficial femoral artery stenting (SFAS), is a preferred method in the treatment of patients with LEAD (3). However, due to bifurcation of common femoral artery caused by some lesions, SFAS frequently has adverse effects on deep femoral artery and is accompanied by in-stent restenosis (4). In these patients, deep femoral artery profundoplasty (DFAP) can improve collateral branches and increase blood supply, but not restore the anatomical structure of the superficial femoral artery (5). Hybrid operations, such as combined SFAS and DFAP, might make the best of both SFAS and DFAP in patients with LEAD (3). However, to date, there have been scarce studies that observe its application in Chinese patients, and there have only been controversial results based on the summary of clinical cases.

China, the largest developing country in the world, has become an aging society, and there are increased morbidity and mortality rates in the elderly (6). LEAD increases sharply with age, and it exponentially increases after the age of 65 (3). There were 6–10% elderly with ischemic symptoms of lower extremity, while 15–20% elderly with asymptomatic ischemia of lower extremity. Clinical data on the application of combined SFAS and DFAP in Chinese patients with LEAD remain scarce, and it is essential to analyze the combination of SFAS and DFAP in these patients classified as Rutherford grades 3–5 (3). Thus, this study aimed to compare the efficiency of simple SFAS with that of combined SFAS and DFAP in the treatment of Chinese patients with LEAD classified as Rutherford grades 3–5.

## Methods

### Study Participants

This study included a total of 200 patients with LEAD classified as Rutherford grades 3–5 who were admitted to Department of Vascular Surgery in our hospital from January 2013 to January 2017. Duplex sonography and computed tomography angiography (CTA) were performed on all the patients after admission (3). In accordance with ESC Guidelines on the diagnosis and treatment of peripheral artery diseases, all the patients were included based on the following criteria: 1) LEAD diagnosed by chief physicians; 2) Rutherford grades 3, 4, and 5; 3) occlusive length of superficial femoral artery >15 cm; 4) initial stenosis of deep femoral artery >30%; 5) stenosis of iliac artery <30%; 6) distal stenosis of popliteal artery <70%; and lastly, 7) stenosis of anterior tibial artery, posterior tibial artery, and peroneal artery <70%. Exclusion criteria as follows: 1) LEAD not caused by arteriosclerosis; 2) Rutherford grades 1, 2, and 6; 3) occlusive length of superficial femoral artery ≤ 15 cm; 4) initial stenosis of deep femoral artery ≤ 30%; 5) stenosis of iliac artery ≥30%; 6) distal stenosis of popliteal artery ≥70%; and lastly, 7) stenosis of anterior tibial artery, posterior tibial artery, and peroneal artery ≥70%.

### Study Procedures

The simple SFAS group included 100 patients, and the combined SFAS and DFAP group included 100 patients. The patients in both groups were allocated by clinical judgment. The simple SFAS group received only SFAS, and the combined SFAS and DFAP group received SFAS and DFAP simultaneously. In the simple SFAS group, modified Seldinger method was applied to puncture the common femoral artery of unaffected extremity. The abdominal aortic and double iliac arteries were radiographed using artery sheath (6 Fr) and pigtail catheter. Long sheath (6–8 Fr) and Cobra Catheter were inserted into the common femoral artery of affected extremity to radiograph superficial femoral and distal arteries. After systemic heparinization, an elbowed catheter and a super smooth guide wire were passed through the occlusive part of the superficial femoral artery, and a Zilver (diameter: 5–6 mm, length: 40–120 mm; Cook Medical, United States) self-expanding stent was placed after balloon dilatation to achieve residual stenosis <30%. There were generally two stents implanted based on the length of vascular lesion. In the combined SFAS and DFAP group, endarterectomy was performed after freeing and carving the initial part of common and deep femoral arteries (5–10 mm), and the Zilver self-expanding stent was placed in the superficial femoral artery after the balloon dilatation. Local vascular condition determined the application of expanded suture with Aesculap (area: 1 cm × 7 cm; Braun, Germany) patch. All the patients received aspirin (100 mg once a day), clopidogrel (100 mg once a day), and low molecular heparin (100 IU/kg once every 12 h). Three days later, the low molecular heparin was changed to cilostazol (100 mg twice per day). Before and 2 days after surgery, ankle brachial index (ABI) was determined by duplex sonography of lower extremity (3). All the patients had a follow-up term of 2 years and had received duplex sonography and CTA in the outpatient department of our hospital. No patient was lost or died after the surgery. The primary end was patency rate, and the second ends were amputation and claudication distance <200 m.

### Statistical Analyses

Continuous variables with normal distribution were reported using mean and standard deviation, and the difference between the two groups was compared by Student's *t*-test. Continuous variables with abnormal distribution were reported using median and interquartile range, and the difference between the two groups was compared by Mann-Whitney *U* test. Categorical variables were reported with number and percentage, and the difference between the two groups was compared by Chi-square test. All analyses were carried out with the SPSS version 18.0 software (SPSS Inc, Chicago, IL, United States), and *P* value <0.05 was accepted as statistically significant.

## Results

All the patients had a median age of 71 years, and there were 143 males (71.5%). Baseline characteristics before surgery, such as age, sex, medical history, clinical appearance, and distal vessels, had no significant difference between the two groups (*P* > 0.05 for all; Table 1). As shown in Table 2, not only increase rate of ABI but also reduction rate of Rutherford grade were significantly higher after surgery in the combined SFAS and DFAP group than in the simple SFAS group (*P* < 0.05 for all). Patency rate of patients in the combined SFAS and DFAP group was significantly higher than that of patients in the simple SFAS group during the follow-up (*P* < 0.05). Proportion of amputation and claudication distance <200 m had no significant difference between the two groups during the follow-up (*P* > 0.05 for all).

**Table 1:** Comparison of baseline characteristics before surgery between the two groups.

**Characteristics**	**Total**	**Simple SFAS**	**Combined SFAS and DFAP**	***P*** **value**
	**(*n* = 200)**	**(*n* = 100)**	**(*n* = 100)**	
Age (years)	71 (68–74)	70 (68–73)	71 (68–74)	0.515
Male (%)	143 (71.5)	69 (69.0)	74 (74.0)	0.434
Smoking (%)	68 (68.0)	31 (31.0)	37 (37.0)	0.370
Medical histories (%)				
Hypertension	149 (74.5)	73 (73.0)	76 (76.0)	0.626
Diabetes mellitus	97 (97)	46 (46.0)	51 (51.0)	0.479
Coronary artery disease	86 (43.0)	41 (41.0)	44 (44.0)	0.568
Clinical appearances (%)				
Intermittent claudication	147 (73.5)	71 (71.0)	76 (76.0)	0.423
Rest pain	117 (58.5)	55 (55.0)	62 (62.0)	0.315
Ulcer and gangrene	35 (17.5)	16 (16.0)	19 (19.0)	0.577
Distal vessels (%)				0.840
Single vessel	103 (51.5)	50 (50.0)	53 (53.0)	
Double vessel	81 (40.5)	41 (41.0)	40 (40.0)	
Triple vessel	16 (8.0)	9 (9.0)	7 (7.0)	

*SFAS, superficial femoral artery stenting; DFAP, deep femoral artery profundoplasty*.

**Table 2:** Comparison of vascular prognosis after surgery between the two groups.

**Characteristics**	**Total**	**Simple SFAS**	**Combined SFAS and DFAP**	***P*** **value**
	**(*n* = 200)**	**(*n* = 100)**	**(*n* = 100)**	
After surgery				
Increase rate of ABI (%)	187 (93.5)	90 (90.0)	97 (97.0)	0.045
Reduction rate of Rutherford grade (%)	183 (91.5)	87 (87.0)	96 (96.0)	0.022
Follow up				
Patency rate (%)	132 (66.0)	59 (59.0)	73 (73.0)	0.037
Amputation (%)	18 (9.0)	11 (11.0)	7 (7.0)	0.323
Claudication distance <200 m (%)	71 (35.5)	39 (39)	32 (32)	0.301

*SFAS, superficial femoral artery stenting; DFAP, deep femoral artery profundoplasty*.

## Discussion

This study demonstrated that compared with SFAS, combined SFAS and DFAP improved not only ABI and Rutherford grade after surgery, but also patency rate during the follow-up in the Chinese patients with LEAD.

Lower extremity artery disease (LEAD) not only severely affects the quality of life of individuals but also leads to economic burden in society (1, 2). LEAD has increased prevalence, and patients with LEAD often have severe lesions, such as long occlusion, multiple calcification, and hard plaques (3). However, due to abundant collateral circulation, they have relatively mild symptoms (7). Only when the initial stenosis of deep femoral artery is >30% or the common femoral artery is involved that the collateral circulation is injured and ischemic symptoms are obvious in patients with LEAD (8).

Both SFAS and DFAP have been gradually applied and have shown certain efficiency in clinical practice (9). In previous studies, SFAS has resulted in obvious improvement of ABI and Rutherford grade after surgery by restoring anatomical structure, but patency rate after SFAS was not very high during the follow-up (10, 11). Intimal hyperplasia and in-stent restenosis are the main problems faced by bare stent in the treatment of LEAD. One study has found that patency rate after SFAS was 64% in 274 patients with LEAD during the follow-up (10). Another study has suggested that patency rate after SFAS was 51% in 432 patients with LEAD during the follow-up (11). Meanwhile, the deep femoral artery has the ability to improve blood circulation in the lower extremities with the help of a vascular network around the knee and hip joints. Main anastomotic branches involved in the vascular network are: 1) ascending branch of the lateral femoral circumflex artery and branches of superior and inferior gluteal arteries; 2) medial femoral circumflex artery and branches of obturator artery; 3) descending branch of the lateral circumflex femoral artery and lateral superior genicular branch of popliteal artery; lastly, 4) perforating artery and uppermost superior genicular branch of the popliteal artery (12). However, in previous studies, although DFAP increased distal blood supply in the lower extremities by improving collateral circulation, its increased blood supply and reduced distal pressure were very limited compared with SFAS (13). Thus, DFAP played certain roles in avoiding amputation and alleviating symptoms, but there was no obvious improvement of ABI and Rutherford grade after DFAP.

On the basis of SFAS, the combined DFAP achieves puncture under direct vision and reduces difficult level of SFAS. On the one hand, combined SFAS and DFAP restores the anatomical structure of the superficial femoral artery and alleviates clinical symptoms at once after surgery. On the other hand, combined SFAS and DFAP improves collateral circulation from the deep femoral artery and vascular patency rate in the long term (3). As a kind of hybrid operation, combined SFAS and DFAP might be a very good surgery method for Chinese patients with LEAD.

This study has several limitations. First, it is a retrospective, non-randomized, and controlled study, which has an effect on its validity. Nevertheless, its results are of great clinical importance. Secondly, lifetime was not obtained in the current study and it is not feasible to perform life-table analysis. Third, the patients in both groups were allocated by clinical judgment; this could be a cause of selection bias in this study.

## Conclusion

This study demonstrated that, compared with SFAS, combined SFAS and DFAP improved not only ABI and Rutherford grade after surgery, but also patency rate during the follow-up in Chinese patients with LEAD. Hybrid operation has a significant value in alleviating clinical symptoms after surgery, thus, improving vascular prognosis in Chinese patients.

## Data Availability Statement

The original contributions presented in the study are included in the article/supplementary material, further inquiries can be directed to the corresponding author/s.

## Ethics Statement

The studies involving human participants were reviewed and approved by Ethics Committee of Aerospace Center Hospital. The patients/participants provided their written informed consent to participate in this study.

## Author Contributions

GC and MD: conceived and designed the study, performed the study, analyzed the data, and wrote the article. All the authors have read and approved the manuscript.

## Conflict of Interest

The authors declare that the research was conducted in the absence of any commercial or financial relationships that could be construed as a potential conflict of interest.

## Publisher's Note

All claims expressed in this article are solely those of the authors and do not necessarily represent those of their affiliated organizations, or those of the publisher, the editors and the reviewers. Any product that may be evaluated in this article, or claim that may be made by its manufacturer, is not guaranteed or endorsed by the publisher.

## Abbreviations

DFAP: deep femoral artery profundoplasty
LEAD: lower extremity artery disease
SFAS: superficial femoral artery stenting.
